# Supplementary material for: HbA1c is inversely associated with thyroid cysts in a euthyroid population: A cross-sectional study
Source: PLoS One. 2021 Jun 30;16(6):e0253841. doi: 10.1371/journal.pone.0253841 (PMC8244890; doi:10.1371/journal.pone.0253841)
Supplement: Attachment — Submitted filename: Reviewers comment(2020.12.21).pdf [file pone.0253841.s001.pdf]

Reviewer #1: The manuscript entitled "HbA1c is inversely associated with thyroid cyst among euthyroid population: a cross sectional study" describes an inverse association between HbA1c levels and thyroid cysts in euthyroid patients. The Authors interpret this finding as a potential tool to explore a reduction in thyroid function.
The idea is original but the manuscript leaves too many edges without a clear explanation.

(Answer.1)
→Thank you for valuable comment. I thought the point that reader feel lack of clear explanation is in our basal hypothesis; absence of thyroid cysts might indicate latent damage of thyroid.

In addition to the fact that the fluid collected from thyroid cysts is reported to be rich in thyroglobulin [Ref1], at that time when I submit present manuscript, only one article which supports above mentioned hypothesis [Ref2]; anti-thyroid peroxidase antibody (TPO-Ab) shows inversely associated with thyroid cysts. Since TPO-Ab is well known cause of autoimmune thyroiditis, participants with higher titer of TPO-Ab might have latent damage in thyroid. And TPO-Ab is significantly inversely associated with thyroid cysts. Then we thought, absence of thyroid cyst might indicate latent damage of thyroid.

However, we published another article during submitting present manuscript that explains thyroid cyst could influence on thyroid function [Ref2]. And also we could publish the article that explain the potential mechanism that underlying the TPO-Ab and subclinical hypothyroidism [Ref3]. In this article, we reported that presence of thyroid cysts has influenced on the association between TPO-Ab and subclinical hypothyroidism. Therefore, we could better explanation now. Then we revised by using those our previous studies as references.

[Ref1]
Salabé GB, et al. Identification of serum proteins, thyroglobulin and antithyroid antibodies in the fluid of thyroid cysts. Thyroidology. 1990;2(1):17-23.
[Ref2]
Shimizu Y, et al. Anti-thyroid peroxidase antibody and thyroid cysts among the general Japanese population: a cross-sectional study. Environ Health Prev Med. 2020;25(1):7.
[Ref3]
Shimizu Y, et al. Association between thyroid-stimulating hormone and hypertension according to thyroid cysts status in the general population: a cross-sectional study.

Environ Health Prev Med. 2020;25(1):69.

[Ref4]

Shimizu Y, et al. Anti-thyroid peroxidase antibody and subclinical hypothyroidism in relation to hypertension and thyroid cysts. PLoS One. 2020;15(10):e0240198.

Because of those reasons, I revised abstract as following. Those sentences explain functional role that might thyroid cyst possess.

> A stronger correlation between thyroid-stimulating hormone (TSH) and free triiodothyronine (T3) was reported among euthyroid participants without thyroid cysts than among those with thyroid cysts. This correlation could be enhanced by low productivity of thyroid hormones. Then, absence of thyroid cysts could indicate low activity of thyroid production.

And I also added the following sentences in introduction section that explain the functional role that might thyroid cyst could have.

> The correlation between thyroid-stimulating hormone (TSH) and free T3 might be enhanced among participants with low thyroid hormone productivity, which is related to latent damage to the thyroid. Previously, we reported that euthyroid individuals without thyroid cysts had a stronger correlation between TSH and free T3 than those with thyroid cysts [3]. Therefore, the absence of thyroid cysts might negatively affect thyroid hormone synthesis.

After describing the potential functional role that thyroid cysts might have, we describe the association between TPO-Ab and thyroid cysts because this association indicates that absence of thyroid cyst could indicate latent damage of thyroid.

And in discussion section, we first described about the latent damage of thyroid that relates to subclinical hypothyroidism. And clarified the association between TPO-Ab and hypothyroidism in relation to thyroid cysts as following.

> A high prevalence of TPO-Ab (+) has been reported in subclinical hypothyroidism [8,9], and hypothyroidism is known to be associated with hypertension [10]. Our previous study also revealed a positive association between subclinical hypothyroidism and hypertension among participants without thyroid cysts but not

among participants with thyroid cysts [7]. Thus, the absence of thyroid cysts might have a disadvantage in the production of thyroid hormones, possibly by indicating latent damage of the thyroid.

In the present study, we found further evidence that HbA1c was significantly inversely associated with thyroid cysts in the euthyroid population (Table 4).

Major comments

1. Introduction: page 6 line 103: without preference data. Do you mean personal habits?

(Answer.2)

➔Thank you for valuable comment. According to this reviewer's valuable comment, I revised as following.

personal habits (smoking status and drinking status)

2. The introduction could benefit of further description on how 2 apparently disconnected parameters such as HbA1c and TPOab are linked. Is the carbohydrate alteration related to thyroid autoimmunity ?

(Answer.3)

➔

Thank you for valuable comment. We reconsider the meaning of anti-thyroid peroxidase antibody (TPO-Ab) in present study. Previously, we reported inverse association between TPO-Ab and thyroid cysts among eu-thyroid population [Ref2]. Since TPO-Ab is known cause of autoimmune thyroiditis that induce thyroid damage, we thought absence of TPO-Ab among eu-thyroid population could indicate latent damage of thyroid. Therefore, the fact that absence of thyroid cyst indicates latent damage of thyroid is important but not TPO-Ab. However, thanks to this reviewer's valuable comment, I recognized that present description could mislead. Then we reconsider the way to describe the fact that absence of thyroid cyst might indicate latent thyroid damage that relates to lower productivity of thyroid hormone.

Previously, we also reported that eu-thyroid participants without thyroid cysts shows stronger correlation between thyroid-stimulating hormone (TSH) and free triiodothyronine (T3) than that of with thyroid cysts [Ref02]. Since this correlation could

be enhanced by low productivity of thyroid hormone, at least partly, absence of thyroid cysts could indicate low activity of thyroid production.

Then, to avoid the misleading, first, we focus on the thyroid cysts status specific association between TSH and free T3 which indicates that absence of thyroid cysts might have disadvantage in producing thyroid hormone. And after that, we also introduce our previous study that shows significant inverse association between thyroid cysts and TPO-Ab among eu-thyroid population because this inverse association also indicates that absence of thyroid cyst among eu-thyroid population might indicates latent damage of thyroid.

In addition to that, even absence of thyroid cysts might indicate latent damage of thyroid [Ref2], the potential influence of thyroid cysts on thyroid function is not yet discussed in introduction section. We also reported in previous study that indicates absence of thyroid cysts could be associated with lower function of thyroid [Ref4].

The specific change in present manuscripts were described for former answer (Answer.1).

3. Considering that thyroid cysts are common and 2 mm is a small size it would be better to clarify what the Authors refer to with "status for cysts" is it "yes or no" or "number of cysts"?

(Answer.4)
→Thank you for valuable comment. According to this reviewer's valuable comment, after edited by native English check we revised as following.

> The presence or absence of thyroid cysts was determined by experienced technicians using LOGIQ Book XP with a 10-MHz transducer (GE Healthcare, Milwaukee, WI, USA). A thyroid cyst was defined to have a maximum diameter of ≥2.0 mm without a solid component, as indicated in our previous study [3,4,7].

4. In statistical methods it would be useful to describe the tests in relation to what is shown later in the results section. In table 1 patients are distributed in 3 groups according to HbA1c levels but this is not introduced in methods. For the logistic regression where does

it say that HbA1c and cysts are going to be analysed as it states in table 3?

(Answer.5)

→Thank you for valuable comment. According to this reviewer's valuable comment, we described as following.

Characteristics of the entire study population as well as the entire population based on HbA1c levels (<5.4% for T1 [low], 5.4-5.6 % for T2 [medium], and 5.7% ≤ for T3 [high]) were expressed as means ± SDs, except for the categories of male sex, use of glucose-lowering medication, preference history (current smoker and daily drinker), and TSH. These categories were expressed as percentages. As TSH showed a skewed distribution, it was expressed as the median (first quartile, third quartile). Significant differences in HbA1c levels were evaluated using analysis of variance.

5. A description of the biochemical and anthropometric parameters of the whole population should be included.

(Answer.6)

→Thank you for valuable comment. According to this reviewer's valuable comment, we added table 1 that shows biochemical and anthropologic parameter of the whole population. And we also added the following sentences in result section.

In the study population, 564 (32.7%) were diagnosed to have thyroid cysts.

Characteristics of the study population

Characteristics of the study population are shown in Table 1. Among the 1,724 patients enrolled, 37.0 % were men. FT3 and FT4 levels in the population were 3.17 ± 0.32 pg/mL and 1.25 ± 0.16 ng/dL, respectively. The median [the first quartile, the third quartile] levels of TSH were 1.58 [1.08, 2.29] μIU/mL.

6. Which is the proportion of cysts according to HbA1c tertiles?

(Answer.7)

→Thank you for valuable comment. According to this reviewer's valuable comment, we

rechecked the present analyzes. Since our present study evaluates the association between HbA1c levels and thyroid cysts, the proportion of cysts according to HbA1c tertiles were shown in tables (Table 4 and Table 5) with description of "No. of cases". To better To better understand, we added following sentence in foot note of table 4 and table 5.

Cases: participants with thyroid cysts.

7. Table 1 please define levels of low, median, high level of HbA1c

(Answer.8)

→Thank you for valuable comment. According to this reviewer's valuable comment, we rechecked the table. Since we already described the exact number of HbA1c tertile levels in footnote of the tables. We added following sentence in first paragraph of the statistical analyzes section.

Characteristics of the entire study population as well as the entire population based on HbA1c levels (<5.4% for T1 [low], 5.4-5.6 % for T2 [medium], and 5.7% ≤ for T3 [high])

8. References are quite scarce considering the abundance on the topic of thyroid and carbohydrate metabolism.

(Answer.9)

→Thank you for valuable comment. As above mentioned reason, we could include recent our reports and strength the background of our hypothesis. In addition to that, thanks to this reviewer's valuable advice we added following sentence.

Hypothyroidism can break the equilibrium between thyroid hormone and glucose homeostasis and alter glucose metabolism, which can lead to insulin resistance [14].

Then the number of references became almost double.

9. The manuscript should be revised for the English language.

Minor comments

1. Several grammar mistakes have to be corrected as in lines 29-31-39-68-71-72-118-132-143-164-165-172-173-194-196-231-254

(Answer.10)

→Thank you for valuable comment. Even before submit this manuscript, our manuscript was edited by professional English editing service, I realized there are many grammatical mistake. I informed this issue to staff of professional English editing service and made them check whole sentences again.

Reviewer #2: This manuscript suffers from many flaws, making it very difficult to read and evaluate. For example, there are several points inconsistent between the text and the tables, the quality of English is very poor, and the meaning of the conclusion is also unclear.

(Answer)

→

Thank you for valuable comment. According to this reviewer's valuable comment, I reconsider the content of present manuscript.

*To clarify the association between HbA1c and thyroid cyst among participants with normal thyroid function is the main purpose of present study because those findings indicates that even participants with latent thyroid damage showed normal range of thyroid hormone, those participants might have the same disadvantage in thyroid hormone activity.

However, before discuss about the above mentioned hypothesis, we have to clarify the fact that a) absence of thyroid cyst could indicate the latent damage of thyroid and b) elevated HbA1c levels could be observed among latent damage of thyroid.

a) About the absence of thyroid cyst as a latent damage of thyroid

Previously, we reported that absence of thyroid cysts could act as an indicator of latent thyroid damage by following three reasons (1~3).

1) Thyroid peroxidase antibody (TPO-Ab) which is well known cause of auto immune thyroiditis is reveled to be significantly inversely associated with thyroid cyst among participants with normal thyroid function [Ref1]. Since TPO-Ab might induce thyroid damage even among participants with normal thyroid function, absence of thyroid cyst could indicate latent damage of thyroid.

[Ref1]

Shimizu Y, et al. Anti-thyroid peroxidase antibody and thyroid cysts among the general Japanese population: a cross-sectional study. Environ Helath Prev Med. 2020;25(1):7.

2) In addition to above mentioned reasons, we also found significant positive association between subclinical hypothyroidism and hypertension among subjects without thyroid cysts but not among subjects with thyroid cysts [Ref2]. Since high prevalence of TPO-Ab is reported in subclinical hypothyroidism [Ref3][Ref4] and hypothyroidism is known to be associated with hypertension [Ref5], absence of thyroid cyst also might have disadvantage in production of thyroid hormone.

[Ref2]

Shimizu T, et al. Anti-thyroid peroxidase antibody and subclinical hypothyroidism in relation to hypertension and thyroid cysts. PLoS One. 2020;15(10):e0240198.

[Ref3]

Darya S, et al. Assessment of subclinical hypothyroidism for a clinical score and thyroid peroxidase antibody: a comparison with euthyroidism grouped by different thyroid-stimulating hormone levels. Asian Biomedicine. 2019;13:85-93.

[Ref4]

Mohanty S, et al. Diagnostic strategies for subclinical hypothyroidism. Indian J Clin Biochem. 2008;23(3):279-282.

[Ref5]

Stabouli S, et al. Hypothyroidism and hypertension. Expert Rev Cardiovasc Ther. 2010;8(11):1559-1565.

3) Furthermore, previously, we found that among eu-thyroid population, subjects without thyroid cysts shows stronger correlation between thyroid stimulating hormone (TSH) and thyroid hormone than that of with thyroid cysts [Ref6]. Since the correlation between thyroid-stimulating hormone (TSH) and free T3 might be enhanced among participants with low thyroid hormone productivity that relates to latent damage of thyroid, absence of thyroid cysts could indicate latent damage of thyroid.

[Ref6]
Shimizu Y, et al. Association between thyroid-stimulating hormone and hypertension according to thyroid cysts status in the general population: a cross-sectional study. Environ Helath Prev Med. 2020;25(1):69.

b) About elevated HbA1c levels could be observed among latent damage of thyroid. Hypothyroidism can break the equilibrium between thyroid hormone and glucose homeostasis and alter glucose metabolism which can lead to insulin resistance [Ref7] and insulin resistance elevates HbA1c level. Then higher HbA1c could be associated with lower activity of thyroid hormone partly by indicating latent damage of thyroid.
[Ref7]
Brenta G. Why can insulin resistance be a natural consequence of thyroid dysfunction? J Thyroid Res. 2011;2011:152850.

Since those manuscripts [Ref2] and [Ref6] were not published when I submit present manuscript, we could not include in text. However, now we can use those references then we revised.

In abstract, we as following that explaining the potential function that thyroid cyst might possess.

A stronger correlation between thyroid-stimulating hormone (TSH) and free triiodothyronine (T3) was reported among euthyroid participants without thyroid cysts than among those with thyroid cysts. This correlation could be enhanced by low productivity of thyroid hormones. Then, absence of thyroid cysts could indicate low activity of thyroid production.

And we added the following sentences in introduction section.

The correlation between thyroid-stimulating hormone (TSH) and free T3 might be enhanced among participants with low thyroid hormone productivity, which is related to latent damage to the thyroid. Previously, we reported that euthyroid individuals without thyroid cysts had a stronger correlation between TSH and free T3 than those with thyroid cysts [3]. Therefore, the absence of thyroid cysts might negatively affect thyroid hormone synthesis.

After describing above mentioned sentences, we described about the association between TPO-Ab and thyroid cysts because presence of TPO-Ab among participants with normal thyroid function could indicate presence of latent thyroid damage. Since subclinical hypothyroidism is one of the condition that is associated with thyroid damage, we described following sentences in discussion section.

> The absence of thyroid cysts might indicate latent damage to the thyroid, as a previous study on a euthyroid population revealed an independent inverse association between thyroid cysts and TPO-Ab, which is a known cause of autoimmune thyroiditis [4].
>
> A high prevalence of TPO-Ab (+) has been reported in subclinical hypothyroidism [8,9], and hypothyroidism is known to be associated with hypertension [10]. Our previous study also revealed a positive association between subclinical hypothyroidism and hypertension among participants without thyroid cysts but not among participants with thyroid cysts [7]. Thus, the absence of thyroid cysts might have a disadvantage in the production of thyroid hormones, possibly by indicating latent damage of the thyroid.

In addition, we described as following sentences that indicates lower activity of thyroid could be associated with insulin resistance.

> Hypothyroidism can break the equilibrium between thyroid hormone and glucose homeostasis and alter glucose metabolism, which can lead to insulin resistance [14].

*To clarify the association between description is text and tables, we added table number in discussion section where sentences is described present results.

* We also added the following sentences that explain why we made further analyzes limited to participants without taking glucose lowering medication. And describe what can be indicating from these additional analyzes.

> Because the same glucose-lowering medication could influence thyroid function [15], which could influence the main results of our study, we conducted further analyses limited to participants who were not receiving glucose-lowering medications. A significant inverse association between HbA1c levels and thyroid

cysts was observed even in the analyses that were limited to participants not receiving glucose-lowering medications (Table 5). Therefore, glucose-lowering medications that influence HbA1c levels could not explain the present main findings.

*Our present manuscript has already edited by professional English editing service. Even though, same grammatical error was pointed. Then we informed this valuable comment to the professional English service and made them editing for all manuscript again.
