# Supplementary material for: HbA1c is inversely associated with thyroid cysts in a euthyroid population: A cross-sectional study
Source: PLoS One. 2021 Jun 30;16(6):e0253841. doi: 10.1371/journal.pone.0253841 (PMC8244890; doi:10.1371/journal.pone.0253841)
Supplement: Attachment — Submitted filename: Comments (2021.1.22).pdf [file pone.0253841.s002.pdf]

Reviewer #2:

1)

I think your conclusion is unreasonable from your results. First, in lines 50-53 in the Abstract, you wrote “Previously, we reported that euthyroid individuals without thyroid cysts had a stronger correlation between TSH and free T3 than those with thyroid cysts [3]. Therefore, the absence of thyroid cysts might negatively affect thyroid hormone synthesis.” However, those correlations were $r=-0.13$ ($p<0.001$) in Thyroid cyst (-) group and $r=-0.03$ ($p=0.525$) in Thyroid cyst (+) group. In lines 232-237 in the Discussion, you wrote “As the absence of thyroid cysts might indicate latent damage to the thyroid [3,4,7], insulin resistance related to TSH levels might underlie the association between thyroid cysts and HbA1c. In fact, in our additional analysis, a slight but significant positive association between TSH and HbA1c levels was observed, while significant inverse associations between TSH and thyroid hormone (free T3 and free T4) levels were observed among all euthyroid participants (Table 3).” However, those results showed $r<0.15$ (absolute value). Your interpretation of those results does not seem appropriate. If a Correlation Coefficient is less than 0.2 (absolute value), it is considered negligible. Even if it has statistical significance, it doesn’t necessarily mean it has medical significance.

→

Thank you for valuable comment. According to this valuable comment, I rechecked the present conclusions and potential mechanism that might underlying present results.

I’m completely agreed with this reviewer’s comment that showed even the statistical power showed the significant value, correlation coefficient less than 0.2 is clinically meaning less.

Therefore, as we described in discussion section that we thought the sensitivity of using the absence of thyroid cysts and higher levels of HbA1c to detect latent damage to thyroid could be much greater than that of using TSH.

To clarify those associations, we added the analysis that clarified the association between HbA1c and subclinical hypothyroidism because latent damage of thyroid might be associated with hypothyroidism.

And we found significant positive association between HbA1c and subclinical hypothyroidism. Therefore, we thought, HbA1c could indicate latent damage of thyroid.

Then we added following sentences in

> Odds ratios (ORs) and 95% confidence intervals (CIs) were calculated using logistic regression models to assess association between HbA1c and subclinical hypothyroidism.

And we also added following sentences in result section with table.

> **Association between subclinical hypothyroidism and HbA1c**
> Table 3 shows a significant positive association between HbA1c and subclinical hypothyroidism that is independent of known cardiovascular risk factors.

We also added following sentences in discussion section.

> In the present study, we found a significant positive association between subclinical hypothyroidism and HbA1c (Table 3). This is consistent with the findings of a previous study that reported the levels of HbA1c in non-diabetic participants with subclinical hypothyroidism to be higher than those in normal healthy controls [7].

However, HbA1c levels is not correlated with thyroid hormone (free T3 and free T4). And even HbA1c shows slightly significantly positively correlated with TSH, the magnitude of this correlation is too small to explain present results. Therefore, we thought the sensitivity of HbA1c that detecting latent damage of thyroid might be stronger than TSH.

Previous study reports that lower thyroid function may lead to extended longevity among elderly [Ref1]. The we thought the demands of thyroid decreased with process of aging. Therefore, not only actual serum concentration of thyroid hormone levels but also demands of thyroid hormone might determine the secretion levels of TSH. However, equilibrium between thyroid hormone and glucose might determine the insulin resistance [Ref2], HbA1c could indicate the deficiency of thyroid hormone.

[Ref1]
Gesing A, Lewiński A, Karbownik-Lewińska M. The thyroid gland and the process of aging; what is new? Thyroid Res. 2012;5(1):16. doi: 10.1186/1756-6614-5-16.
[Ref2]

Brenta G. Why can insulin resistance be a natural consequence of thyroid dysfunction? J Thyroid Res. 2011;2011:152850.

Then we added following sentences that explains potential mechanism of present results in discussion section.

The magnitude of the observed associations (between TSH and HbA1c, and between TSH and thyroid hormones) were too small to explain the present results showing a significant inverse association between HbA1c and thyroid cysts. The magnitude of physiological demand for thyroid hormone activity might explain this discrepancy. Because decreased thyroid function may extend longevity, the demand for thyroid hormone activity may decrease with age [16]. Therefore, not only the serum thyroid hormone level, but also the demand for thyroid activity might determine the level of TSH secretion. HbA1c levels may indicate the magnitude of thyroid hormone deficiency in the same way that the equilibrium between thyroid hormone and glucose might determine insulin resistance [15]. In the present study, HbA1c was significantly positively correlated with subclinical hypothyroidism (Table 3) but not with thyroid hormone (Table 4). Because the absence of thyroid hormone indicates latent damage to the thyroid [3,5,8], HbA1c levels could inversely associate with thyroid cysts (Table 5).

The same associations between HbA1c levels and thyroid cysts were observed when analyses were limited to the normal TSH range (Table 5).

Therefore, the sensitivity of using the absence of thyroid cysts and higher levels of HbA1c to detect latent damage to the thyroid could be much greater than that of using TSH level.

And we deleted the description of the correlation among thyroid cysts, thyroid hormone and TSH in abstract section and introduction section.

Furthermore, we added the following sentence in introduction section that partly indicates that subjects with thyroid cyst could possess higher activity of thyroid hormone.

We previously identified a significant positive association between thyroid cysts and isolated systolic hypertension [3]. Since hyperthyroidism is known to be a common cause of isolated systolic hypertension [4], development of thyroid cysts could benefit patients with low thyroid hormone synthesis, and absence of thyroid cysts might attenuate thyroid hormone synthesis.

And we added the definition of subclinical hypothyroidism as following.

The threshold for subclinical hypothyroidism is set at TSH > 4.01 μIU/mL.

2)

The odds ratio in Table 5 for Models 1, 2, 3 are around 0.75. Those don't seem to indicate strong associations. Based on the above points, I think you are jumping to the conclusion that your findings provide an efficient tool to evaluate the thyroid hormone activity that relates to insulin resistance.

→

Thank you for valuable comment. Since most of those subjects showed normal range of HbA1c, we revised to use the clinical cut off values of HbA1c. And we found that compared to the subjects with completely normal range of HbA1c (<5.6%), subjects with range of diabetes (6.5% ≤ HbA1c) shows 0.48 (0.28, 0.81). When we excluded the subjects with taking glucose lowering medication, the value became 0.38 (0.16, 0.88). Therefore, HbA1c might strongly inversely associated with thyroid cysts.

3)

Furthermore, in line 119, you wrote "… to determine the association between TPO-Ab and thyroid cysts", but you didn't measure TPO-Ab. Therefore, you cannot determine that association. That kind of statement is confusing for the reader.

→

This is completely typo. Sorry for inconvenience. I corrected.

Reviewer #3: Revisions are extremely helpful. The English is now clear and the import of the report is evident.

→

Thank you for valuable comment.
