# Supplementary material for: HbA1c is inversely associated with thyroid cysts in a euthyroid population: A cross-sectional study
Source: PLoS One. 2021 Jun 30;16(6):e0253841. doi: 10.1371/journal.pone.0253841 (PMC8244890; doi:10.1371/journal.pone.0253841)
Supplement: Attachment — Submitted filename: Comments (2021.3.22).pdf [file pone.0253841.s003.pdf]

Reviewer #2: Thank you for the revision. The results were interesting. It might be valuable for publication. However, I still don’t agree with your conclusion.
In the discussion, you wrote “the absence of thyroid cysts might indicate latent damage to the thyroid" (line 223) and “which could not be evaluated based on TSH, free T3 and free T4 levels" (lines 269-270). It sounds like you are suggesting a hypothesis that the absence of thyroid cysts could be used to diagnose latent damage and thyroid activity.
I could understand if you said the existence of thyroid cysts might indicate a high level of thyroid activity. However, it is difficult to say the absence of thyroid cysts indicate latent damage. Among the people without thyroid cysts, there must be a lot of people with completely healthy thyroids. Isn’t it more appropriate to think there is only a small fraction of people with latent damage? Jumping to conclusions like that will lead to mistaken diagnoses.

→
(Reply)
Thank you for your valuable comment. According to this valuable comment, we reconsider the scientific meaning of present results.

As shown in Table 3, we found significant positive association between HbA1c and subclinical hypothyroidisms. This indicates that, high level of HbA1c is associated with subclinical thyroid disorder.

And as shown in Table 5, we found significant inverse association between HbA1c and thyroid cysts.

Then in present study, high level of HbA1c that could be associated with subclinical thyroid disorder revealed to be inversely associated with thyroid cysts. This is why we thought absence of thyroid cyst might indicate latent thyroid damage that is associated with high levels of HbA1c (insulin resistance).

In order to show the scientific evidence that supports “existence of thyroid cysts might indicate a high levels of activity” as this reviewer commented, we have to perform other analyzes that showed two associations. The first one is an inverse association between HbA1c and subclinical hyperthyroidism. And second one is a positive association between HbA1c and thyroid cysts.

Even HbA1c shows positively associated with subclinical hypothyroidism as shown in Table 2, this fact never indicate that HbA1c is inversely associated with subclinical hyperthyroidism. In order to describe about the influence of higher activity of thyroid hormone, we should show significant inverse association between subclinical hyperthyroidism and HbA1c.

In addition to that, even hypothyroidism is reported to be associated with diabetes [Ref1], another study also reports that not only serum TSH levels but also T3 levels were increased in participants with diabetes [Ref2]. This means both of hyperthyroidism and hypothyroidism might increase the level of HbA1c. Therefore, positive association between HbA1c and thyroid cysts should be observed if high levels of thyroid hormone activity that associated with thyroid cysts causes main results of present study. However, we found significant inverse association between HbA1c and thyroid cysts.

[Ref1]
Makada MG et al. Study of glycated haemoglobin (HbA1c) in non-diabetic subjects with subclinical hypothyroidism. J Clin Diagn Res. 2017;11(4):BC01-04.
[Ref2]
Elgazar FH, et al. Thyroid dysfunction prevalence and relation to glycemic control in patients with type 2 diabetes mellitus. Diabetes Metab Syndr. 2019;13(4):2513-2517.

This is why we can’t describe as existence of thyroid cysts might reduce the HbA1c in present study by indicating a high level activity of thyroid hormone. Then we did describes as following in discussion section.

> Both hyperthyroidism and hypothyroidism are reported to be associated with the development of insulin resistance [12]. However, a positive linear association between the normal range of TSH level and insulin resistance among non-diabetes participants and type 2 diabetes patients has been reported [13]. Furthermore, a positive correlation between HbA1c and TSH levels was also observed in patients with subclinical hypothyroidism [14].

Even though, I also could understand the meaning that this reviewer thought that there are too many completely healthy participants without thyroid cysts.

However, we also have to regard the fact that we have no efficient diagnostic tool to

evaluate the latent thyroid damage that could not be evaluated by thyroid hormones and thyroid stimulating hormone.

Then we could not figure out the exact prevalence rate of participants with completely healthy thyroid. In fact, there is growing evidence suggesting that the treatment of subclinical hypothyroidism may not be beneficial, particularly in older patients [Ref3]. However, aging patients may progress from subclinical hypothyroidism to overt hypothyroidism. Therefore, determining the timing to begin treatment for hypothyroidism is difficult in daily clinical practice [Ref4]. This difficulty might partly be caused by latent damage of thyroid and decreased demand of thyroid activity along with process of aging.

[Ref3]
Leng O, Razvi S. Hypothyroidism in the older population. Thyroid Res. 2019;12.2 doi: 10.1186/s13044-019-0063-3.
[Ref4]
Khandelwal D, Tandon N. Overt and subclinical hypothyroidism: who to treat and how. Drugs. 2012;72(1):17-33.

This is why we described the sentence “at least partly” as following in abstract.

> Therefore, at least partly, absence of thyroid cysts could indicate latent thyroid damage.

And we did describe the potential function that thyroid cyst might possesses as following in introduction section.

> We previously identified a significant positive association between thyroid cysts and isolated systolic hypertension [3]. Since hyperthyroidism is known to be a common cause of isolated systolic hypertension [4], development of thyroid cysts could benefit patients with low thyroid hormone synthesis, and absence of thyroid cysts might attenuate thyroid hormone synthesis.

This description is also important because this sentences indicates that thyroid cysts can support thyroid hormone synthesis but not activates aggressive thyroid hormone synthesis. And we described why absence of thyroid cysts could be associated with

latent thyroid damage as following in introduction section.

> As TPO-Ab inhibits thyroid peroxidase, which plays an important role in synthesizing thyroid hormones [6], the absence of thyroid cysts in euthyroid individuals could indicate latent damage to the thyroid.

Even this reviewer commented as “It sound like you are suggesting a hypothesis that the absence of thyroid cysts could be used to diagnose latent damage and thyroid activity.”, we never thought that the presence of latent thyroid damage could be estimated only by absence of thyroid cysts. This is why we did describe as following in discussion section.

> the sensitivity of using the absence of thyroid cysts and higher levels of HbA1c to detect latent damage to the thyroid could be much greater than that of using TSH level.

And because our present study is not intended to describe that HbA1c is efficient tool to evaluate the latent damage of thyroid but to describe that status of thyroid cysts could be influence on hormone activity that relates to insulin resistance. Then we describe in conclusion as “Although further investigation on the present topic are necessary, the present finding provides an efficient tool to evaluate the thyroid hormone activity relative to insulin resistance among participants with normal thyroid function.

Because of those above mentioned reasons, I’m disagree with this reviewer’s valuable comment.

#3 Reviewer: Previously, anti-peroxidase antibodies (TPO-Ab), which are known cause autoimmune thyroiditis, were shown to be present in individuals in inverse proportion to the prevalence of thyroid cysts.

□→(Reply)

Thank you for valuable comment. According to this reviewer's valuable comment, we re-checked the content of present study. Since we evaluated the existence of a thyroid cysts on the parameter whether it was present or not, we did not evaluate the association between the number of thyroid cysts (size of the thyroid cysts) and HbA1c. This is important fact that should be taken into consideration. Therefore, we thought the sentence "were shown to be present in individuals in inverse proportion to the prevalence of thyroid cysts" might not appropriate to use. Then we described as following.

> Previously, anti-peroxidase antibody (TPO-Ab) which is known cause of autoimmune thyroiditis is revealed to be inversely associated with thyroid cysts among euthyroid population.

□→(Reply)

Thank you for valuable comment. According to this reviewer's valuable comment, we re-checked the content of present study. Since present study is a cross-sectional study, we could not determine the causal relationships. Therefore, the sentence "incidence of" is not appropriate to use. Then we revised as following.

> Since participants with subclinical hypothyroidism are reported to have higher HbA1c than normal healthy controls, HbA1c could be inversely associated with presence of thyroid cysts through a mechanism reflecting latent thyroid damage.
