# Supplementary material for: HbA1c is inversely associated with thyroid cysts in a euthyroid population: A cross-sectional study
Source: PLoS One. 2021 Jun 30;16(6):e0253841. doi: 10.1371/journal.pone.0253841 (PMC8244890; doi:10.1371/journal.pone.0253841)
Supplement: Attachment — Submitted filename: Rebuttal.PLoS One(2021.6.3).pdf [file pone.0253841.s006.pdf]

Reviewer #1: All comments have been adequately answered
I am still in doubt if the p values in table 6 are correct since they are non significant.
→
Thank you for your valuable comment. In present study, we categorized HbA1c as <5.6%, 5.6-6.4%, and 6.5%≤. The analysis that is shown in Table 6 is limited to participants without taking glucose lowering medication. Then the number of participants with normal range of HbA1c is much higher than that of high HbA1c level. Among not taking glucose lowering medication, the number of participant (case of thyroid cysts) in each category are 983 (319), 584 (205) and 52 (10). Then those categories are biased in number. That results in p>0.05 level when considering trends as shown in table 6. Even thought, when we made those analyzes by using tertile categories of HbA1c, the number of participants and thyroid cases were 543 (176) for T1, 530 (188) for T2 and 434 (136) for T3. And the p-values were 0.021 for Model 1, 0.025 for Model 2 and 0.0446 for Model 3. Therefore, the findings that this reviewer pointed out is the matter of distribution. Possibly, the wide range of 95% CI and Odds shows nearly 1 for subjects with HbA1c (5.6-6.4%), might results in no significant values when considering p as trend.

Reviewer #2: Thank you for the revision. Since you deleted the sentence, "Therefore, the sensitivity of using the absence of thyroid cysts and higher levels of HbA1c to detect latent damage to the thyroid could be much greater than that of using TSH level.", I am able to accept your logic a little. However, I still disagree with your conclusion. In the conclusion, you wrote, "the present finding provides an efficient tool to evaluate the thyroid hormone activity relative to insulin resistance among participants with normal thyroid function." However, this conclusion was based on your hypothesis, not your results.

You should put, "The absence of thyroid cysts and higher levels of HbA1c could indicate the latent functional damage of the thyroid" into the Conclusion, as you wrote in the Discussion, and delete "the present finding provides an efficient tool to evaluate

the thyroid hormone activity relative to insulin resistance among participants with normal thyroid function.” The same is true about the Abstract.

→

Thank you for your valuable comment. According to this valuable comment, I deleted the sentence “the present finding provides an efficient tool to evaluate the thyroid hormone activity relative to insulin resistance among participants with normal thyroid function” from conclusion section of abstract and discussion. And described as “The absence of thyroid cysts and higher levels of HbA1c could indicate the latent functional damage of the thyroid”.

Furthermore, in lines 274-275 in the Discussion, you wrote, “the present study also provides an efficient tool to evaluate the validity of HbA1c level control in participants with normal thyroid function.” I think you should also change this sentence for the same reason.

→

Thank you for your valuable comment. According to this valuable comment, I deleted the sentence “he present study also provides an efficient tool to evaluate the validity of HbA1c level control in participants with normal thyroid function.” Since the last sentence indicates the importance of Hba1c level control in subjects with subclinical hypothyroid, we added the sentence “Present findings could provide efficient cues to control HbA1c associated with the latent functional damage of the thyroid.”
