# Supplementary material for: HbA1c is inversely associated with thyroid cysts in a euthyroid population: A cross-sectional study
Source: PLoS One. 2021 Jun 30;16(6):e0253841. doi: 10.1371/journal.pone.0253841 (PMC8244890; doi:10.1371/journal.pone.0253841)
Supplement: Attachment — Submitted filename: Dear Editor-in-Chief_PONE-D-20-13979R3.docx [file pone.0253841.s004.docx]

**Manuscript Number PONE-D-20-13979R3**

Dear Editor-in-Chief,

I think the results of this study are interesting. If you want to follow up on this manuscript, please find another reviewer.

Mihoko Takahashi
