# Supplementary material for: HbA1c is inversely associated with thyroid cysts in a euthyroid population: A cross-sectional study
Source: PLoS One. 2021 Jun 30;16(6):e0253841. doi: 10.1371/journal.pone.0253841 (PMC8244890; doi:10.1371/journal.pone.0253841)
Supplement: Attachment — Submitted filename: Rebuttal (2021.4.30)PLoS One.docx [file pone.0253841.s005.docx]

**Reviewer #1:** Even though the manuscript has been improved there are still some concerns with the meaning of some sentences that would need to be modified.

1)Line 20
“Previously, anti-peroxidase antibody (TPO-Ab) which is known cause of autoimmune
thyroiditis is revealed to be inversely…”
I would suggest rephrasing the following sentences for clarity.
→

Thank you for your valuable comment. According to this valuable comment, we revised as following.

Anti-peroxidase antibody (TPO-Ab) is revealed to be inversely associated with thyroid cysts among euthyroid population. TPO-Ab causes autoimmune thyroiditis by bolstering thyroid inflammation.

2) Line 38
“investigations are necessary, the present findings help efficiently evaluate thyroid
hormone activity corresponding to insulin resistance among participants with normal…”
Please rephrase since the meaning of the sentence is not clear
→

Thank you for your valuable comment. According to this valuable comment, we reconsider the meaning of this sentence. Since the fact that further investigation is necessary on present topic are the things to taken for granted, we delete the sentence. And simplify as following.

Present finding provides an efficient tool to evaluate the thyroid hormone activity relative to insulin resistance among participants with normal thyroid function.

3) Line 49
“and isolated systolic hypertension [3]. Since hyperthyroidism is known to be a common
cause of isolated systolic hypertension [4], development of thyroid cysts could benefit
patients with low thyroid hormone synthesis, and absence of thyroid cysts might
attenuate thyroid hormone synthesis.”
I understand the Authors want to state that the absence of thyroid cysts might reflect attenuated thyroid hormone synthesis but the whole sentence does not make sense . Please rephrase.
→

Thank you for your valuable comment. According to this valuable comment, we reconsider the meaning of this sentence. Hyperthyroidism is known major secondary causes of isolated systolic hypertension. And because we found significant positive association between thyroid cysts and isolated hypertension in our previous study with euthyroid participants, we could described that euthyriod participants with thyroid cyst might have relatively higher thyroid hormone synthesizing activity than those without thyroid cysts. But the sentence that “the absence of thyroid cysts might reflect attenuated thyroid hormone synthesis” is not appropriate. Since we described that the absence of thyroid cysts in euthyroid individuals could indicate latent damage to the thyroid in next section, we revised as following.

We previously identified a significant positive association between thyroid cysts and isolated systolic hypertension among euthyroid participants [3]. Since hyperthyroidism is a major secondary cause of isolated hypertension [4], euthyroid participants with thyroid cysts might have relatively higher thyroid hormone synthesizing activity than those without thyroid cysts.

4) Line 110
6.5≤ Does it means less than 6.5?
→

Thank you for your valuable comment. According to this valuable comment, we recheck the relevant sentence. This is HbA1c level (<5.6% for [low], 5.6-6.4 % for [medium], and 6.5% ≤ for [high]). Then meaning of 6.5% ≤ was Hba1c levels as and above 6.5%. In tabled we also described as <5.6%, 5.6-6.4 % , and 6.5% ≤ ). Therefore, <5.6% means less than 5.6%. To avoid misunderstand, we added the word [low] [medium] and [high] for tables.

5)Footnote table 3: please correct “HDLc”
→

Thank you for your valuable comment. According to this valuable comment, we recheck the relevant sentence. Sorry for inconvenience, I corrected.

6) Title table 6: please correct “without taking glucose lowering”
→

Thank you for your valuable comment. According to this valuable comment, we recheck the relevant sentence. Since we used as “not receiving glucose-lowering medication” we revised as so.

7)Line 255: “Therefore, the sensitivity of using the absence of thyroid cysts and higher levels of
HbA1c to detect latent damage to the thyroid could be much greater than that of using
TSH level.”
I would suggest changing this sentence because the sensitivity of these putative diagnostic tools has not been assessed in this study and cannot replace a standardized biochemical test such as TSH that has been traditionally used to detect hypothyroidism.

→

Thank you for your valuable comment. According to this valuable comment, we reconsider the relevant sentence. And I revised as following,

Therefore, the absence of thyroid cysts and higher levels of HbA1c could indicate the latent functional damage of the thyroid.

**Reviewer #2:** Thank you for the reply.
I read this manuscript again after reading your explanation. However, my concern still remains. In fact, you wrote “I also could understand the meaning that this reviewer thought that there are too many completely healthy participants without thyroid cysts.” However, you didn’t discuss that in the manuscript. Thus, you didn’t reflect my comments at all, even though you agree with part of my comments, nor did you provide a satisfactory explanation for the part of my comments with which you disagreed. Consequently, I recommend rejecting this manuscript.

→

Thank you for your valuable comment. Even this reviewer thought that there is no description about the completely healthy participants without thyroid cysts, we already described those sentences with closed meaning as I made reply before. Those sentences describe that the thyroid cysts might have the clinical feature on thyroid function and absence of thyroid hormone might have disadvantage on synthesizing thyroid hormone with same references.

In addition to that, we also described about the age-related reduction of demand of thyroid hormone as following.

However, the magnitude of the observed associations (between TSH and HbA1c, and between TSH and thyroid hormones) were too small to explain the present results showing a significant inverse association between HbA1c and thyroid cysts. The magnitude of physiological demand for thyroid hormone activity might explain this discrepancy. Because decreased thyroid function may extend longevity, the demand for thyroid hormone activity may decrease with age [16]. Therefore, not only the serum thyroid hormone level, but also the demand for thyroid activity might determine the level of TSH secretion.

Those description indicates that, diagnosing as completely healthy thyroid condition only by thyroid hormone level which is not considering about age-related demand level is inappropriate. And making criteria to diagnose completely healthy thyroid is not main topic of present study. Then we reconsider what sentence could be added without deteriorating scientific meaning to this review’s comment. To diagnose as having completely healthy thyroid condition among euthyroid individuals, at least we should evaluate the turnover of thyroid hormone which is not common for annual health check-up and daily clinical practice but needs the technique of nuclear medicine. Since we could not evaluate the completely healthy condition and the word “completely healthy condition” is not scientific, we added following sentences in limitation section.

Third, the turnover of the thyroid hormone may be influenced by status of thyroid cysts, but we could not evaluate the half-life of thyroid hormone.

**Reviewer #3:** The manuscript reads well and is of considerable interest.

→

Thank you for your valuable comment.
